# Legionnaires’ Disease Presenting With Acute Kidney Injury: Successful Treatment With Antibiotics

**DOI:** 10.7759/cureus.74360

**Published:** 2024-11-24

**Authors:** Ai Fujii, Mizuki Mishima, Sho Kumano, Keiji Fujimoto, Kengo Furuichi

**Affiliations:** 1 Nephrology, Kanazawa Medical University, Kahoku, JPN; 2 Internal Medicine, Keiju Medical Center, Nanao, JPN

**Keywords:** acute kidney injury, acute tubular necrosis (atn), anti-biotics, kidney biopsy, legionnaires' disease

## Abstract

Legionnaires' disease is a bacterial infection caused by *Legionella*, such as *Legionella pneumophila*. It mainly causes severe pneumonia, with symptoms such as fever, cough, and shortness of breath. In rare cases, it can cause acute kidney disease and also occasionally become severe enough to require replacement therapy. We report the case of a patient who was admitted with severe pneumonitis and acute kidney injury following *Legionella* infection. Symptoms improved with antibiotic treatment; however, renal damage persisted. A renal biopsy was performed, highlighting the importance of recognizing kidney involvement in *Legionella* infections and demonstrating how renal biopsy can guide the course of treatment.

## Introduction

*Legionella pneumophila* has become widely recognized as a cause of community-acquired pneumonia (CAP). Recent studies on severe community-acquired pneumonia have shown that *L. pneumophila* is the second most common cause of ICU admissions [1]. It has been associated with various complications, including renal involvement. This often presents as mild, transient azotemia, hematuria, proteinuria, or pyuria and some patients have required hemodialysis [2]. Despite numerous case reports, it remains unclear whether *L. pneumophila* directly causes acute kidney disease. The most important feature of *Legionella* infection is that they are intracellular proliferating bacteria. In nature, it proliferates primarily within amoeba, and in humans, it primarily proliferates within white blood cells (macrophages). However, it has also been reported to infect cells in various organs, including the kidneys. Here, we report a case of acute kidney injury (AKI) with oliguria after *Legionella* infection followed by a renal biopsy [1]. We consider this an important case in understanding kidney damage caused by *Legionella*.

## Case presentation

A 51-year-old man was transferred to our hospital with severe pneumonitis and AKI. The patient had a fever (maximum temperature of 38°C), cough, and fatigue for the past seven days. His symptoms persisted and he visited the emergency room of a local hospital two days prior to his presentation where his serum creatine was 6.24 mg/dL (normal range: 0.7-1.3 mg/dL). He remained oliguric even after a 500 ml intravenous extracellular fluid drip. During the treatment at the emergency room, he did not show any signs of vital shock or low blood pressure. He was transferred to our hospital due to severe pneumonia and AKI with oliguria. He was only taking medication for hyperlipidemia. He never smoked. There was no family history for autoimmune or chronic kidney diseases.

**Figure 1:**
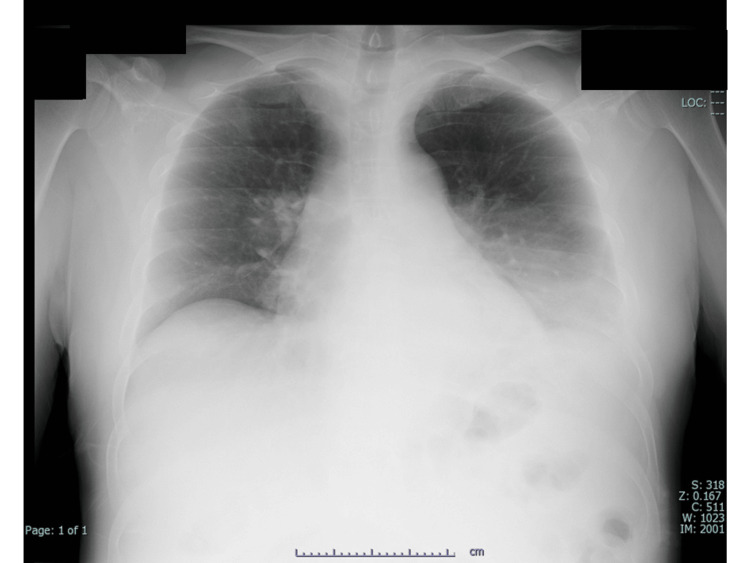
Chest X-ray showing bilateral lower lung consolidation.

**Figure 2:**
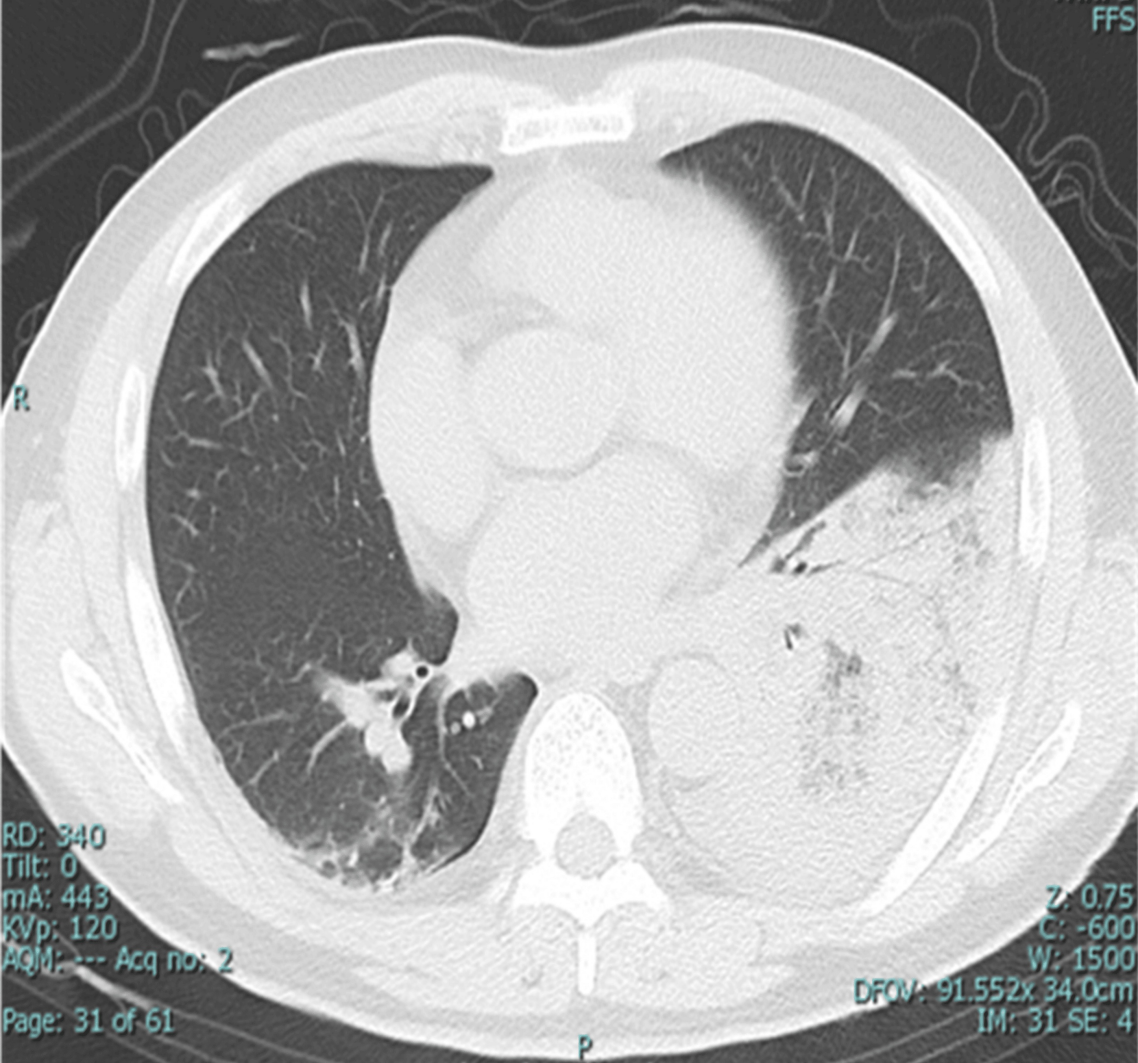
CT of chest revealing diffuse bilateral consolidation opacities in the lower lobes.

After the transfer to our hospital, he had no fever, his heart rate was 65 beats per minute, and his blood pressure was 116/80 mmHg. There was no muscle pain. His oxygen saturation was 93% in room air. Blood gas analysis showed pH 7.437, partial pressure of oxygen (PO2) 70 mmHg, and partial pressure of carbon dioxide (PCO2) 30 mmHg. Moist crackles were detected on both dorsal back sides. Chest X-ray showed bilateral lower lung consolidation (Figure 1), and CT revealed diffuse bilateral consolidation opacities in the lower lobes (Figure 2).

Laboratory data on admission revealed normal white blood cells (7010/μL, 82% neutrophils, 7% lymphocytes), hematocrit 36.9%, hyponatremia (sodium (Na) 132 mmol/L), and elevated C-reactive protein (CRP) levels (25.22 mg/dL) and procalcitonin (2.56 ng/mL). The remaining data showed serum creatinine 7.6 mg/dL, blood urea 50 mg/dL, aspartate aminotransferase (AST) 169 IU, alanine transaminase (ALT) 158 IU, creatine phosphokinase (CPK) 87 IU/L, and lactate dehydrogenase (LDH) 384 IU/L (Table 1). Urinalysis revealed 1+ proteinuria and ± hematuria, and a urinary *Legionella* antigen test was positive. The patient's blood cultures and viral serology (Epstein-Barr virus (EBV), cytomegalovirus (CMV), hepatitis C virus (HCV), hepatitis B virus (HBV), and HIV) were negative.

**Table 1:** Changes in laboratory values during the clinical course of two hospitalizations Ht: hematocrit; Hb: hemoglobin; PLT: platelet count; SGOT: serum glutamic-oxaloacetic transaminase; AST: aspartate aminotransferase; SGPT: serum glutamic pyruvic transaminase; ALT: alanine transaminase; LDH: lactate dehydrogenase; CPK: creatine phosphokinase; ALP: alkaline phosphatase; CRP: C-reactive protein; γGTP/GTT: gamma-glutamyl transpeptidase

	1st admission Day 0	2nd admission Day 1	Day 7	Day 13	Day 23	Day 80
WBC (K/μL)	9300	7010	6590	5000	4690	5020
Ht/Hb (%, gr/dL)	37.4/13.3	36.9/12.4	33.7/11	33.6/11.4	33.1/10.9	39.6/12.8
PLT (K/μL)	15.9	18.3	38.5	27.8	24.4	27
SGOT/AST (U/L)	128	169	31	21	14	16
SGPT/ALT (U/L)	156	158	52	26	12	13
LDH (U/L)	389	384	251	198	155	153
CPK (U/L)	88	87	22	28	70	68
ALP (U/L)	310	442	241	151	77	62
γGTP/GTT (U/L)	290	353	214	132	43	18
Total proteins/albumin (gr/dL)	6.0/3.5	5.0/2.0	5.6/2.7	6.2/3.4	6.6/4.3	6.5/4.2
Urea (mg/dL)	41.2	50	35	21	18	18
Creatinine (mg/dL)	6.24	7.6	3.51	1.76	1.29	1.27
Potassium (mEq/L)	130	132	141	140	143	142
Sodium (mEq/L)	3.5	3.4	3.6	4.1	4.3	4.4
Calcium(mg/dL)	8.6	10.1	10.1	9.6	9.3	9.3
Phosphorus (mg/dL)	3.4	4.9	4.3	3.1	3.6	3.9
CRP (mg/dL)	35.75	25.22	0.79	0.13	0.02	0.02

Due to an elevated white blood cell count with a predominance of neutrophils and high levels of procalcitonin, combined antibiotic treatment (levofloxacin (LVFX) and ceftriaxone (CTRX)) was started. Over the next few days, his fever, respiratory symptoms, and radiological findings improved. Laboratory markers of inflammation and renal function gradually improved.

**Figure 3:**
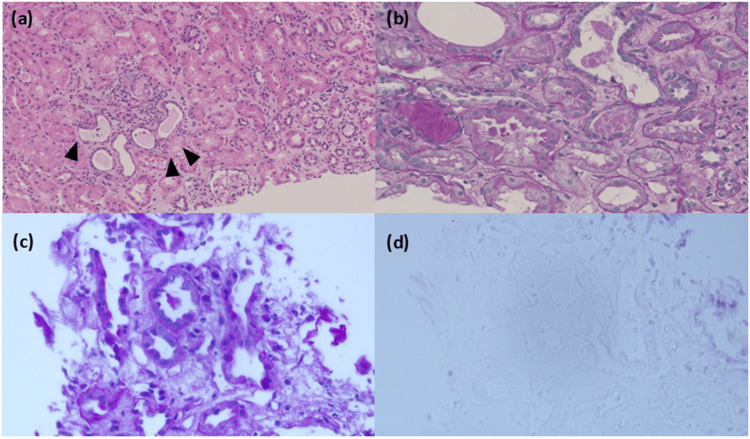
Renal biopsy. (a) Findings from light microscopy showed interstitial cell infiltrate associated with edema and few tubules lined by flattened cells (arrow head, H&E stain). The glomeruli didn’t have abnormalities of the glomerular basement membrane; (b) A characteristic feature of nephrotoxicity was desquamated cell fragments and bleb formation in the renal tubules (periodic acid-Schiff stain). No bacteria were detected in the tissue by comparing the periodic acid-Schiff stain (c) to the Gimenez stain (d) in the same view.

On the 14th day of hospitalization, a kidney biopsy was performed. Light microscopy findings revealed eight glomeruli that seemed to be almost normal. In the tubulointerstitium, an inflammatory cell infiltration (mainly lymphocytes) was observed. Moreover, patchy or diffuse shedding of renal tubular cells from the tubular basement membrane, tubular dilation, and flattening of renal tubular cells were observed (Figure 3). The findings of the kidney biopsy were diagnosed as acute tubular necrosis due to Legionnaires' disease. We performed Gimenez staining to detect *Legionella* bacteria. However, there weren’t any bacteria bodies in the kidney biopsy specimens.

## Discussion

In this case, *Legionella* infection showed pneumonia and AKI. Both pneumonia and AKI improved with antibiotic administration. A kidney biopsy revealed no glomerulonephritis but there was a mild-to-moderate degree of mixed inflammatory cell infiltration, composed of polymorphonuclear leukocytes, lymphocytes in zonal areas of the tubulointerstitium with patchy tubular epithelial cell flattening and atrophy.

Several case reports have revealed that *Legionella* infection can cause renal dysfunction [3]. It is known that rhabdomyolysis is a complication of *Legionella* infection, which can induce AKI [4]. Additionally, AKI occurs in 33-50% of cases of rhabdomyolysis caused by *Legionella *pneumonia [5]. A combination of myoglobin cast obstruction in the distal tubule and tubulointerstitial nephritis has been observed [6,7]. However, in the present case, there were no findings of rhabdomyolysis or myoglobin casts. Mechanisms other than rhabdomyolysis such as intoxication, drug toxicity, and hypotension have also been reported [8]. However, such a clinical condition was not observed in this case. The other mechanisms are very rare, but direct damage from *Legionella* is possible.

*Legionella* is characterized by intracellular infection, and it mainly infects white blood cells in humans. However, it has been suggested that the kidney cell may be the target cell for direct infection and the presence of *Legionella* in kidney tissue has been proven by electron microscopy and immunohistochemistry [8,9]. Gimenez staining is particularly effective in visualizing *Legionella* because it highlights intracellular bacteria. It uses basic fuchsin, which stains the bacteria bright red or pink, making them stand out clearly. We used a Gimenez staining kit (41241, Muto Pure Chemical Co. Ltd., Bunkyo City, Japan), but we didn’t detect the bacteria body in the kidney biopsy specimens. Identifying *Legionella *with Gimenez staining can be challenging because the sensitivity of bacteria body detection in the tubular cells is low. Moreover, we had used antibiotics at the time of kidney biopsy. This could be a reason why we failed to detect *Legionella* in the tubular cells with Gimenez staining. The AKI in this case was speculated to be tubular necrosis due to direct damage to renal tubular cells by *Legionella*, and antibiotic administration was an effective treatment.

In this case, the patient showed pneumonia and AKI due to *Legionella* infection. *Legionella* pneumonia is often accompanied by damage to multiple organs and affects the gastrointestinal system, kidneys, and central nervous system [10]. Multiorgan damage also greatly affects the prognosis of *Legionella *infection. The overall mortality rate for Legionnaires' disease is approximately 15%, but when renal failure is present, the mortality rate increases significantly to 53% [9]. Neurological symptoms are also often observed. It has been reported that the complication rate of neurological symptoms in *Legionella* pneumonia is 40-50%. In addition to immunological mechanisms, direct infection has also been reported as the mechanism of the onset of neurological symptoms [11]. In this case, pulmonary and renal symptoms were observed, but no central nervous system symptoms or gastrointestinal symptoms were observed. The organ specificity of infection is unknown and remains a future issue.

## Conclusions

*Legionella* infection can lead to both pneumonia and AKI, and both conditions improve with antibiotic treatment. It is important to understand how kidney injury, particularly with tubular necrosis, is induced by *Legionella* infection. Additionally, infection control with antibiotic therapy plays a crucial role in recovery.
